# Association between neutrophil to lymphocyte ratio and erectile dysfunction among US males: a population-based cross-sectional study

**DOI:** 10.3389/fendo.2023.1192113

**Published:** 2023-06-23

**Authors:** Xingliang Feng, Yangyang Mei, Xiaogang Wang, Li Cui, Renfang Xu

**Affiliations:** ^1^ Department of Urology, The Third Affiliated Hospital of Soochow University, Changzhou, Jiangsu, China; ^2^ Department of Urology, First People’s Hospital of Changzhou, Changzhou, Jiangsu, China; ^3^ Department of Urology, Jiangyin People’s Hospital of Jiangsu Province, Jiangyin, China

**Keywords:** neutrophil to lymphocyte ratio, NHANES, erectile dysfunction, cross-sectional study, inflammation

## Abstract

**Objective:**

The purpose of the study was to investigate the relationship between neutrophil-to-lymphocyte ratio (NLR) and erectile dysfunction (ED) in adult American males using a large database.

**Methods:**

We adopted a series of statistical analyses of the relationship between NLR indices and ED prevalence among participants in the 2001-2004 National Health and Nutrition Examination Survey (NHANES) database using the R software.

**Results:**

The study included a total of 3012 participants, of whom 570 (18.9%) presented with ED. NLR levels were 2.13 (95% CI: 2.08,2.17) in those without ED and 2.36 (95% CI: 2.27,2.45) in those with ED. After adjusting for confounding variables, NLR levels were higher in patients with ED, (β, 1.21, 95% CI, 1.09-1.34, P < 0.001). In addition, a U-shaped relationship between NLR and ED was observed after controlling for all confounders. A more significant correlation (β, 1.35, 95% CI, 1.19 to 1.53, P < 0.001) existed to the right of the inflection point (1.52).

**Conclusion:**

The results of the large cross-sectional study showed a statistically significant association between the occurrence of ED and NLR, a simple, inexpensive, and readily available parameter of inflammation, in US adults. Further studies are still needed in the future to validate and replicate our findings and to investigate the specific mechanisms involved.

## 1 Introduction

Erectile dysfunction (ED) was defined as the inability to achieve or maintain an erection sufficient for satisfactory sexual intercourse, according to the International Medical Association (1). And ED can have negative impacts on both the physical and mental health of men, as well as their partners’ quality of life, even if it’s not life-threatening (2). A large follow-up study in Massachusetts reported a crude prevalence of ED of 26/1000 person-years (3), and it’s expected that the worldwide rate of ED prevalence will increase rapidly with the rise of comorbidities associated with ED, potentially affecting about 320 million men worldwide by 2025 (4). The etiology of ED is complex, and the current research suggests that it may result from a multifactorial process involving vascular, hormonal, neurological, and anatomical factors (5). It has been reported that the presence and severe degree of ED are related to markers of inflammation and endothelial dysfunction (6). The neutrophil-to-lymphocyte ratio (NLR) has recently been recognized as a prospective biological marker of a generalized inflammatory state with the advantage of convenience and inexpensive and has been reported to have prognostic value in several diseases (7–9). However, only a limited number of studies have investigated the relationship between NLR and ED in the population, and most of them are from Asia, with inconsistent results. A relevant meta-analysis revealed that the NLR was higher in ED patients than in the healthy subjects, but only seven studies were included, and there was significant heterogeneity (10). Additional research is needed due to the slightly smaller sample size of previous studies and the limited adjustment for confounding variables. We hypothesize that there is a potential association between NLR and ED. In the study, we aimed to determine the relations between NLR and ED by using a large database, incorporating more comprehensive population data, and adjusting for confounders as much as possible. Our findings will contribute to a better understanding of the mechanisms of inflammation and exploration of valuable biomarkers.

## 2 Materials and methods

### 2.1 Study population

We obtained relevant data for this study from the National Health and Nutrition Examination Survey (NHANES) database, which is conducted by the National Center for Health Statistics (NCHS), a division of the Centers for Disease Control and Prevention (CDC). The NHANES database uses a complex, probability-based sampling design to assess the health and nutritional status of noninstitutionalized civilians in the United States through standardized interviews, physical examinations, and laboratory tests, providing information from diverse populations (11). The data have been available for research since 1999 and have been issued every two years. For this study, we collected data from the two NHANES cycles (2001-2002, 2003-2004), with more information on the data available on the NHANES website (www.cdc.gov/nchs/nhanes/).

The data sets from two NHANES research cycles (2001-2002 and 2003-2004) were selected for cross-sectional analysis, as ED and NLR index values were only available for these two cycles. From 2001 to 2004, a total of 21161 individuals participated in NHANES. Exclusion criteria were as follows: female (n=10860); missing information on ED (n=6185); age >70 years (n=747); missing information on education level (n=1); missing information on marriage (n=2); missing information on NLR index (n=109); missing information on household income (n=186); missing information on smoking (n=3); missing Alcohol information (n=5); missing BMI information (n=50); missing coronary artery disease (n=1). Finally, a total of 3012 cases were included in this study, including 570 ED patients and 2442 controls.

### 2.2 Data collection and definition

For the assessment of ED, participants were asked to evaluate their competence to achieve and maintain an erection sufficient to enable sexual intercourse in the 2001-2004 information collection, and the response options were “never”, “sometimes”, “usually”, and “almost often or almost always”, and we classified subjects who answered “never” or “sometimes” as individuals with ED. In the sensitivity analysis, only men who selected “never” were considered to suffer from ED (12). Based on these, we started a correlation analysis to uncover the factors associated with ED. The target variable for our primary study was NLR, which was analyzed for neutrophil and lymphocyte counting by whole blood count from a Beckman Coulter automated analyzer. Detailed analysis procedures are described in Chapter 7 of the NHANES Laboratory/Medical Technician Procedures Manual (https://wwwn.cdc.gov/nchs/nhanes/).

Covariates including age, BMI, race, marital status, education, alcohol consumption status, poverty income ratio, smoking status, history of diabetes, and history of hypertension were selected for analysis. The results of BMI were divided into three groups: BMI ≤ 25, 25 < BMI ≤ 30, and BMI > 30. Race was classified as Mexican American, Non-Hispanic White, Non-Hispanic Black, Other Hispanic, and other races. Educational attainment was divided into less than high school, high school, and high school or higher. The marital status was divided into: married/cohabiting with partner and living alone. The poverty income ratio (PIR) is an index of the household income to poverty ratio, which reflects social economic status. These guidelines are published annually by the Department of Health and Human Services (HHS) and are categorized as PIR ≤ 1.3, 1.3 < PIR ≤ 3.5, and PIR > 3.5. These covariates were considered potential confounders that may affect the relationship between NLR and ED and were included in the multivariate model.

### 2.3 Statistical analysis

Since NHANES performs a complex multistage sampling design for the American population, to avoid obtaining unrealistic statistical results, we applied information on the sample weights, subgroups, and substrata to all analyses of the statistics thus enabling the accurate assessment of the included population as much as possible. Weights for the combined survey periods were obtained by dividing the weight of each 2-year period by 2 based on the analysis rules of NHANES (13). Using the survey design R package in R programming, we provided weights to characterize the demographic and clinical parameters of all participants according to the presence or absence of ED in the subject population. Means and standard errors (SE) were used for continuous variables, and frequencies and percentages were used for categorical variables. To analyze the differences between the two groups, we performed linear regression (continuous variables) and chi-square tests (categorical variables). We conducted a cointegration test to eliminate any issues and filtered out valid covariates according to guidelines (14). Further, multiple models were run to adjust for potential confounders and to compare coefficients across adjusted models. In Model 1, no variables were adjusted. Model 2 adjusted for age, marital status, race, and educational attainment. The third model adjusted for alcohol consumption status, smoking, diabetes, hypertension, cardiovascular disease (CVD), PIR, and BMI based on model 2. We further assessed the association between the NLR index and ED with smooth curve fitting (penalized spline method) and generalized additive model regression (GAM). If a non-linear relationship was observed, a dichotomous linear regression method model was used to calculate the threshold effect of NLR. In addition, we assessed multicollinearity with the Variance Inflation Factor (VIF) for all variables, and covariates were excluded if VIF was > 5, indicating cointegration problems. When a nonlinear association was found, we performed a likelihood ratio test to find the inflection point value. Most past studies on the relationship between NLR and ED have not been adjusted for clinical conditions, such as hypertension, diabetes, and cardiovascular disease. Some researchers have suggested adjusting for clinical conditions or health markers to avoid methodological inconsistencies that could affect the reproducibility of the work (15). Therefore, in sensitivity analyses, we did not exclude patients with a history of hypertension, diabetes, or CVD and were stratified by age, race, education level, BMI, diabetes, hypertension, and CVD in the final model. All analyses were performed with the R version 4.2.0 package. All significance tests were two-tailed, and the significance level was set at P < 0.05.

## 3 Results

### 3.1 Characteristics of participants

**Figure 1:**
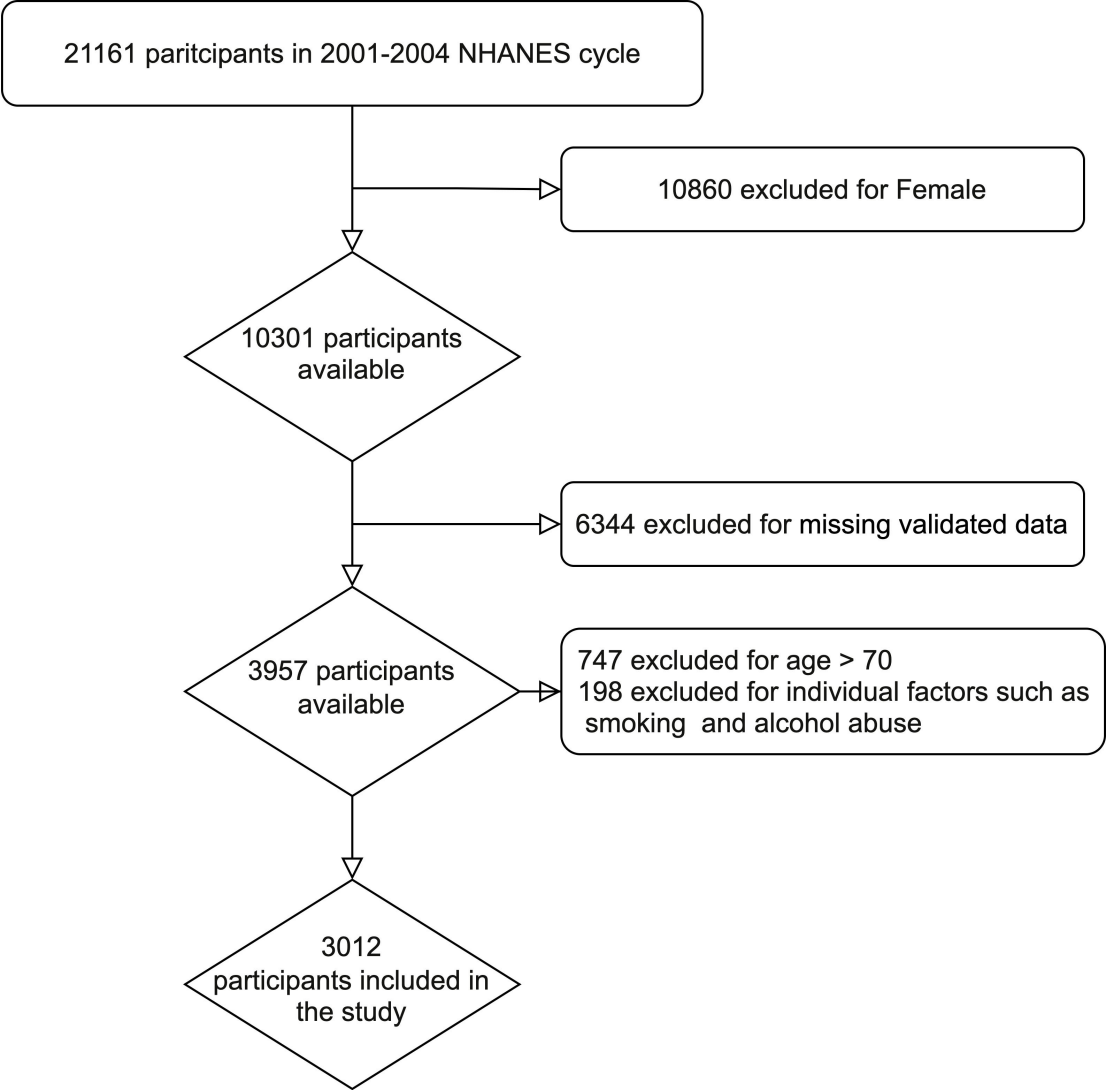
Flow chart of the study.

A total of 21,161 individuals were included in the two NHANES cycles conducted between 2001 and 2004. Following the exclusion criteria described in the Methods, 3012 participants were identified for the study, out of which 570 (18.9%) had ED. The process flow diagram for the specific selection of study participants is shown in **Figure 1**. The baseline characteristics of the included population and the weighted analysis of the study population characteristics for the total sample are detailed in **Table 1**. The levels of NLR were 2.13 (95% CI: 2.08,2.17) in those without ED and 2.36 (95% CI: 2.27,2.45) in those with ED, and the levels of NLR were higher in those with ED, p<0.001. Moreover, the ED group exhibited higher rates of age, BMI, smoking, diabetes, CVD, and hypertension, while education levels and PIR levels were significantly lower. Additionally, rates of being married or cohabiting with a partner were higher in those with ED.

**Table 1:** Baseline characteristics of study participants in NHANES 2001–2004, weighted.

Characteristics	History of erectile dysfunction (ED)		P-value
	No	Yes	
Number (n)	2442	570	
Age, year	40.26 (39.67,40.85)	53.55 (52.41,54.68)	<0.0001
BMI (kg/m^2^), n (%)			<0.0001
BMI≤25	30.67 (28.67,32.74)	22.07 (17.71,27.16)	
25<BMI≤30	41.17 (38.93,43,47)	37.92 (33.43,42.64)	
BMI>30	28.15 (25.85,30.58)	40.00 (34.86,45.37)	
Race, n (%)			0.2544
Mexican American	8.28 (6.40,10.66)	8.99 (5.62,14.07)	
Other Hispanic	4.03 (2.56,6.29)	6.57 (2.82,14.58)	
Non-Hispanic White	73.56 (69.26,77.46)	70.95 (62.52,78.15)	
Non-Hispanic Black	9.75 (7.75,12.19)	9.58 (6.86,13.23)	
Other races	4.38 (3.20,5.96)	3.91 (2.21,6.82)	
Educational level, n (%)			<0.0001
Below high school	13.58 (12.25,15.03)	27.87 (22.44,34.03)	
High school	27.92 (25.49,30.50)	23.30 (19.35,27.79)	
Above high school	58.49 (55.80,61.14)	48.83 (43.41,54.27)	
Marital status, n (%)			<0.0001
Married or living with a partner	68.86 (65.79,71.77)	79.46 (75.38,83.03)	
Living alone	31.14 (28.23,34.21)	20.54 (16.97,24.64)	
PIR, n (%)			0.0053
PIR≤1.3	16.01 (13.97,18.28)	20.62 (16.11,26.00)	
1.3<PIR≤3.5	33.52 (30.89,36.25)	36.40 (31.79,41.27)	
PIR>3.5	50.47 (47.04,53.90)	42.99 (37.69,48.46)	
Alcohol intake, n (%)			0.8229
No	6.96 (4.13,11.49)	6.66 (4.11,10.61)	
Yes	93.05 (88.51,95.88)	93.34 (89.39,95.89)	
Smoking, n (%)			<0.0001
No	45.54 (42.44,48.67)	30.53 (26.33,35.07)	
Yes	54.46 (51.33,57.57)	69.47 (64.93,73.67)	
History of diabetes, n (%)			<0.0001
No	94.61 (93.45,95.57)	71.06 (66.87,74.92)	
Yes	5.39 (4.43,6.55)	28.94 (25.08,33.13)	
History of CVD, n (%)			
No	95.71 (94.62,96.49)	81.43 (76.10,85.79)	<0.0001
Yes	4.29 (3.41,5.39)	18.57 (14.21,23.90)	
History of hypertension, n (%)			
No	71.28 (68.80,73.63)	44.18 (38.86,49.63)	<0.0001
Yes	28.72 (26.37,31.20)	55.83 (50.37,61.14)	
NLR	2.13 (2.08,2.17)	2.36 (2.27,2.45)	0.0003

BMI, body mass index; PIR, poverty income ratio; CVD, cardiovascular disease; NLR, neutrophil to lymphocyte ratio.

For continuous variables: survey-weighted mean (95% CI), P-value was by survey-weighted linear regression.

For categorical variables: survey-weighted percentage (95% CI), P-value was by survey-weighted Chi-square test.

### 3.2 The relationship between NLR and ED

**Figure 2:**
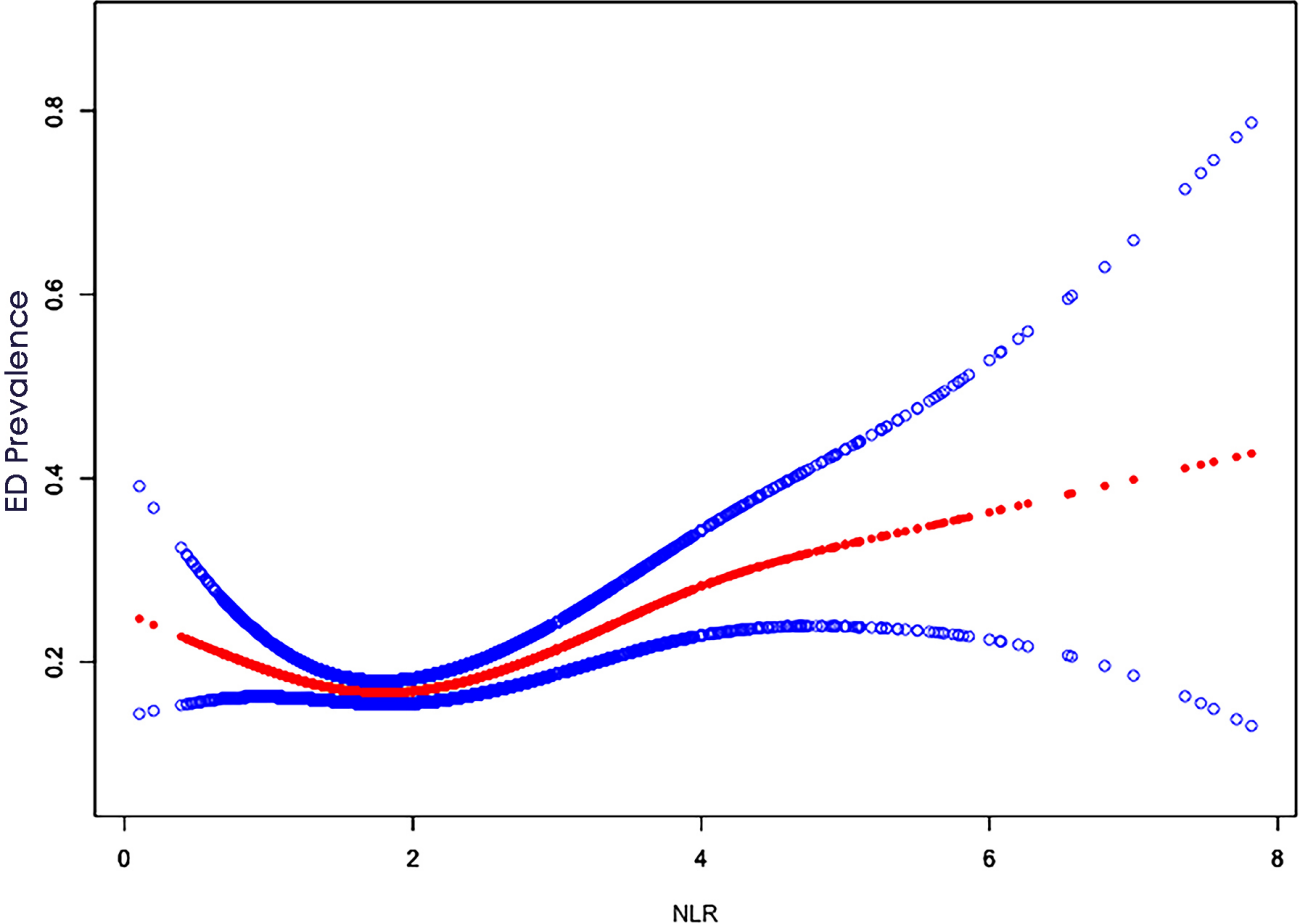
Non-linear relationship plot between NLR and ED.

The detailed relationship between NLR as a continuous variable or quartile of a categorical variable and ED is presented in **Table 2**. In the crude model, NLR was positively associated with severity (β, 1.26, 95% CI, 1.16-1.38, P<0.001). After adjusting for age, race, education, and marital status (Model I), the results did not change significantly (β, 1.214, 95% CI, 1.10,1.35, P < 0.001). Even after adjusting for all covariates (Model III), a significant association between NLR and ED was still observed (β, 1.21, 95% CI, 1.09-1.34, P < 0.001). When NLR was considered as a categorical variable (quartiles), in the crude model, only the population in the Q4 (>2.57) interval was statistically significant (β, 1.50, 95% CI, 1.17-1.93, p=0.001) compared to Q1 (<1.47), while Q2 (1.47-1.94) and Q3 (1.95-2.56) were not statistically significant. In Model II and in the fully adjusted model, Q2, Q3, and Q4 were not statistically significant compared to Q1. In addition, a U-shaped relationship between NLR and ED was observed after adjusting for all covariates (**Figure 2**). With the two-piecewise linear regression model, we found an inflection point of 1.52 (**Table 3**). Although on both sides of the inflection point, there is a positive correlation, the correlation is obviously higher on the right side (β, 1.35, 95% CI, 1.19 to 1.53, P < 0.001) than on the left side (β, 0.46, 95% CI, 0.26 to 0.81, P < 0.01).

**Table 2:** Multivariable logistic regression analyses for NLR and ED, weighted.

Exposure	Crude Model		Adjusted Model 1		Adjusted Model 2	
	OR (95%CI)	P value	OR (95%CI)	P value	OR (95%CI)	P value
NLR (continuous)	1.26 (1.16,1.38)	<0.0001	1.214 (1.10,1.35)	0.0002	1.21 (1.09,1.34)	0.0005
NLR (quartile)						
Q1 (<1.47)	1.0		1.0		1.0	
Q2 (1.47-1.94)	0.86 (0.65.1.13)	0.27	0.77 (0.57,1.04)	0.09	0.74 (0.54,1.02)	0.06
Q3 (1.95-2.56)	1.02 (0.78,1.32)	0.91	0.89 (0.66,1.21)	0.46	0.84 (0.62.1.15)	0.28
Q4 (≥2.57)	1.50 (1.17,1.93)	0.001	1.29 (0.96,1.72)	0.09	1.26 (0.94,1.70)	0.12
P for trend	<0.001		0.03		0.05	

NLR, neutrophil to lymphocyte ratio; ED, erectile dysfunction.

Crude Model: no covariates were adjusted.

Model 2: age, race, education and marital status were adjusted.

Model 3: Model 2+alcohol use, smoking, diabetes, hypertension, CVD, PIR and BMI were adjusted.

**Table 3:** Threshold effect analysis for NLR and ED.

Outcome	β (95%CI)	P value
Fitting model by standard linear regression	1.21 (1.09,1.34)	0.0005
Fitting model by two-piecewise linear regression Inflection point		
<1.52	0.46 (0.26,0.81)	0.0069
≥1.52	1.35 (1.19,1.53)	<0.0001
P for log likelihood ratio test		<0.0001

### 3.3 Subgroup analysis

Further analyses of subgroups were performed according to various confounding factors, as detailed in **Table 4**, showing that age >50 years (OR = 1.34, 95% CI: 1.17, 1.54), Mexican American (OR = 1.34, 95% CI: 1.07, 1.69) and Non-Hispanic White (OR = 1.27, 95% CI: 1.09, 1.47), Below high school (OR = 1.26, 95% CI: 1.05, 1.50) and High school (OR = 1.32, 95% CI: 1.05, 1.66), and BMI between 25 and 30 (OR = 1.27, 95% CI: 1.08, 1.50) subgroups were at higher risk of ED. Furthermore, all subgroups analyzed were examined for interaction, and no statistically significant association was found (P > 0.05 for the interaction).

**Table 4:** Subgroup analysis for NLR and ED, weighted.

Characteristics	Crude model	Adjusted Model 1	Adjusted Model 2	P for interaction
	OR (95%CI)	OR (95%CI)	OR (95%CI)	
Age				0.049
Age≤50y	1.07 (0.89,1.28)	1.07 (0.89,1.29)	1.05 (0.87,1.27)	
Age>50y	1.29 (1.14,1.46)	1.36 (1.19,1.54)	1.34 (1.17,1.53)	
Race				0.11
Mexican American	1.52 (1.25,1.85)	1.37 (1.11,1.69)	1.34 (1.07,1.69)	
Other Hispanic	0.90 (0.56,1.44)	0.87 (0.52-1.44)	0.69 (0.40,1.20)	
Non-Hispanic White	1.35 (1.19,1.53)	1.27 (1.10,1.47)	1.27 (1.09,1.47)	
Non-Hispanic Black	1.09 (0.87,1.36)	1.09 (0.84,1.42)	1.08 (0.83,1.42)	
Other races	0.80 (0.45,1.42)	0.78 (0.42,1.45)	0.82 (0.43,1.56)	
Educational level, n (%)				0.43
Below high school	1.28 (1.09,1.50)	1.21 (1.02,1.44)	1.26 (1.05,1.50)	
High school	1.35 (1.12,1.64)	1.33 (1.07,1.66)	1.32 (1.05,1.66)	
Above high school	1.25 (1.09,1.43)	1.16 (0.99,1.37)	1.11 (0.94,1.32)	
BMI				0.68
BMI≤25	1.19 (1.00,1.41)	1.14 (0.94,1.40)	1.16 (0.96,1.41)	
25<BMI≤30	1.33 (1.15,1.54)	1.24 (1.05,1.46)	1.27 (1.08,1.50)	
BMI>30	1.29 (1.10,1.51)	1.21 (1.01,1.47)	1.15 (0.95,1.40)	
History of diabetes				0.49
No	1.28 (1.15,1.42)	1.23 (1.09,1.38)	1.22 (1.08,1.38)	
Yes	1.19 (0.97,1.47)	1.11 (0.88,1.40)	1.12 (0.89,1.40)	
History of Hypertension				0.53
No	1.22 (1.07,1.40)	1.17 (1.01,1.35)	1.17 (1.01,1.36)	
Yes	1.27 (1.12,1.45)	1.25 (1.08,1.46)	1.25 (1.07,1.46)	
History of CVD				0.15
No	1.26 (1.14,1.39)	1.19 (1.07,1.33)	1.19 (1.06,1.33)	
Yes	1.27 (0.96,1.67)	1.52 (1.08,2.13)	1.55 (1.09,2.20)	

BMI, body mass index; PIR, poverty income ratio; CVD, cardiovascular disease; NLR, neutrophil to lymphocyte ratio.

The subgroup analysis was stratified by age, race, educational level, BMI, diabetes, Hypertension, and CVD, not adjusted for the stratification variable itself.

Crude Model: no covariates were adjusted.

Model 2: age, race, education and marital status were adjusted.

Model 3: Model 2+alcohol use, smoking, diabetes, hypertension, CVD, PIR and BMI were adjusted.

## 4 Discussion

In this large cross-sectional study, we investigated the relationship between ED and NLR levels in American adult males using the NHANES database. After adjusting for appropriate skewed variables, we found a significant association between high levels of NLR and a higher prevalence of ED. Furthermore, we can still observe a clearly existing association after analysis by subgroups. So far as we know, this would be the first research to be performed examining the relationship between the NLR index and ED in a large population through the NHANES database.

With the increasing prevalence of ED, the erectile function of men has received increasing concerns. According to large-scale surveys, older men with ED show a higher frequency of comorbidities with diseases or conditions such as CVD, diabetes, obesity, lower urinary symptoms, and which have been considered risk factors for ED (16, 17). Our study revealed a significantly higher prevalence of CVD in the ED group compared to the control group. And endothelial damage plays a substantial contribution in the development of ED and CVD (18–20). Several studies have suggested that the pathogenesis and severe degree of ED is related to an increase in inflammatory markers, and that lower-grade subclinical inflammation may affect endothelial function and lead to thrombosis. It was reported that there was an increased formation of inflammatory mediators (interleukin (IL)-1β, TNF-α, IL-6, CRP, IL-10), markers, and endokines in patients with ED (21, 22). NLR is a novel inflammatory marker that has drawn the attention of many scholars. Demirkol et al. demonstrated that NLR levels were elevated significantly in patients suffering from cardiac syndrome and CAD. Moreover, they proved a statistically significant association between carotid intima-media thickness and NLR (23). Sambel et al. suggested that the NLR is associated with the diagnosis of ED and that the index is available easily without additional charges (24). Based on these foundations, we aimed to study the relationship between ED and NLR in a sizeable group of American adult men. Our findings revealed that the NLR levels in the subjects group were significantly higher than those in the control group, and there was a significant positive correlation between NLR at more than 1.52 (inflection point) and ED.

Previous studies have demonstrated a range of associations between erectile dysfunction (ED) and various indicators, such as age, BMI, smoking frequency, hypertension, diabetes, CVD, and some inflammatory indices like leukocytes and CRP (25, 26). Recently, the NLR index, derived from routine blood neutrophils and lymphocytes, has emerged as an intuitive and reliable predictor of inflammation levels that contributes to clinical decision-making (12). In our study, age, BMI, smoking prevalence, diabetes, CVD, and hypertension factors were observed to be correlated with an increased prevalence of ED by subgroup analysis. Besides, Mexican-American, non-Hispanic white, low-economic level patients and low-education (below high school and high school) subgroups were found to be at higher risk for ED. It has been previously found that Mexican-American men have a higher prevalence of ED (27), and a correlation between low-economic status and the risk of ED occurrence has also been proposed (28). Similar results have been reported from the NHSLS data, where they first observed an association between income in and ED in the NHSLS sample. The lower the education level, the higher the probability of ED, although the association was not statistically significant. However, such an association was not adjusted for the comorbidities and lifestyle risk factors (29). A more comprehensive study reported an association between ED and education level and occupation, and after adjusting for all risk factors, only occupation had a statistically significant association with ED, which was a higher risk of ED in blue-collar men compared to white-collar men (30), unfortunately, they did not include income as a variable. Results from the MARSH research similarly indicated that higher levels of education were associated with lower odds of ED (31).

In addition to the well-established association between ED and traditional cardiovascular risk factors such as obesity, hypertension, smoking, and diabetes, many authors have found a close link between ED and factors related to CVD (25, 32). This suggests that ED may serve as an early warning sign for CVD. And these risk factors can lead to endothelial dysfunction and eventually to atherosclerosis. The degree of impact from atherosclerosis is similar for all vessels, but the appearance of symptoms varies depending on the diameter of the affected artery (33–35). Since the penile arteries have a smaller diameter (1-2 mm) compared to coronary arteries (3-4 mm), the same extent of endothelial dysfunction and atherosclerosis is more likely to result in a significant decrease in blood flow to the penile tissue at an early stage (33, 35). It has been suggested that the development of atherosclerosis is an active process of inflammation instead of passive damage to blood vessels resulting from lipid infiltration (36, 37). Several clinical studies have shown that inflammation plays a key role in the development and progression of atherosclerosis and can even transform stable atherosclerotic lesions into unstable plaques (37). Besides, subclinical inflammation at a lower level may impair endothelial function and trigger thrombotic events. Therefore, inflammation likely contributes significantly to the progression of ED. There are many researches have investigated the role of NLR in the development of CVD. Considering NLR as a novel marker of inflammation levels, Kalay et al. (38) suggested that NLR levels are elevated markedly in patients with atherosclerosis, and can serve as a biomarker for the development of atherosclerosis.

The normal vascular endothelium is typically resistant to inflammatory properties; however, under conditions of inflammation and increased oxidative stress, the endothelial function can be impaired (39). Moreover, it has been demonstrated that inflammatory stimulation may cause acute or chronic damage to arterial function to some extent (40–43). Notably, plasma levels of C-reactive protein (CRP) were found to be statistically higher in patients with ED who were matched with age and coronary risk scores compared to subjects without ED (21). Besides, in men with ED without clinically significant CVD, CRP levels correlated significantly with the severity of disease of the penile arteries as assessed by penile Doppler ultrasonography (44). In a primary research, increasing levels of fibrinogen were found in ED patients when compared to men with a normal erectile function (45). Some studies have also found that ED is associated with an increased state of inflammation in males presenting with obesity syndrome or metabolic disorders (46, 47). However, it should be emphasized that while the findings of the aforementioned observational and cross-sectional studies are important, they do not necessarily prove a causal relationship. While the penile vessels can be targets of extensive inflammation originating elsewhere, the organ itself may contribute to the general development of inflammation. The male’s corpus cavernosum acts as a paracrine system for the production of angiotensin II (48), and studies have shown that deletion polymorphisms in the gene encoding angiotensin-converting enzyme are more common in men with organic ED (49). Angiotensin II contributes to inflammation in blood vessels by causing oxidative stress and modulating the distribution of inflammatory mediators such as IL-6 (50). It also enhances the expression of adhesion molecules and increases the infiltration of monocytes/macrophages into the vascular wall (51). Although many studies have indicated a relationship between ED and inflammation, the relationship between the two is complex, and the specific causal and pathological mechanisms remain to be further explored.

NLR is a simple, inexpensive, and accessible inflammatory parameter with high sensitivity and low specificity. It can detect dynamic changes in NLR levels before clinical manifestations occur, providing clinicians with early warning signs of an ongoing pathological process. NLR is a novel marker of cellular immune activation and a validated indicator of systemic inflammation, which can open up a new dimension in clinical medicine (52). Based on the relationship between ED and inflammation, we found an association between NLR levels and ED in our study as well, which means that NLR may also be applied in the initial evaluation of ED patients.

However, there are several limitations to our study. First, it is not permissible to draw causal inferences due to the design of the cross-section. Besides, the NLR data came from only an individual blood test and it would be more accurate to evaluate the chronic inflammatory status of the subjects by repeating the test multiple times. Additionally, the findings of the study were acquired in an American population and cannot be generalized to other races. Larger studies in multiracial populations may be more helpful in the future. In the meantime, there are several strengths of the study. First, it is based on a large-scale sample size with a complex survey design that provides a good overview of the US population. Next, we included other confounding factors such as age, cardiovascular history, and economic status that have not been adjusted for concurrently in earlier studies. Finally, the large sample size allowed us to conduct subgroup analyses without significantly reducing statistical power.

In conclusion, the results of this large cross-sectional study suggest a significant association between high levels of NLR and ED in US adults. We were able to observe a clear correlation between the two after subgroup analysis. The positive association between NLR and ED was more apparent when NLR was higher than 1.52. In the future, more research is still needed to verify and replicate our findings and examine the specific mechanisms.

## Data availability statement

The raw data supporting the conclusions of this article will be made available by the authors, without undue reservation.

## Ethics statement

Ethical review and approval was not required for the study on human participants in accordance with the local legislation and institutional requirements. The patients/participants provided their written informed consent to participate in this study. Written informed consent was obtained from the individual(s) for the publication of any potentially identifiable images or data included in this article.

## Author contributions

Data analysis and manuscript writing: XF, YM, and LC; Study design and statistical advice: XF, YM, XW, LC, and RX; Manuscript editing: XF, YM, XW, LC, and RX; Validation and review: LC, and RX; Quality control: XF. All authors contributed to the article and approved the submitted version.
